# Challenging Case of Ectopic ACTH Secretion from Prostate Adenocarcinoma

**DOI:** 10.1155/2022/3739957

**Published:** 2022-03-31

**Authors:** Wanling Zeng, Joan Khoo

**Affiliations:** Department of Endocrinology, Changi General Hospital, 2 Simei Street 3 529889, Singapore

## Abstract

Cushing's syndrome (CS) secondary to ectopic adrenocorticotrophic hormone (ACTH)-producing prostate cancer is rare with less than 50 cases reported. The diagnosis can be challenging due to atypical and variable clinical presentations of this uncommon source of ectopic ACTH secretion. We report a case of Cushing's syndrome secondary to prostate adenocarcinoma who presented with symptoms of severe hypercortisolism with recurrent hypokalaemia, limb oedema, limb weakness, and sepsis. He presented with severe hypokalaemia and metabolic alkalosis (potassium 2.5 mmol/L and bicarbonate 36 mmol/L), with elevated 8 am cortisol 1229 nmol/L. ACTH-dependent Cushing's syndrome was diagnosed with inappropriately normal ACTH 57.4 ng/L, significantly elevated 24-hour urine free cortisol and unsuppressed cortisol after 1 mg low-dose, 2-day low-dose, and 8 mg high-dose dexamethasone suppression tests. ^68^Ga-DOTANOC PET/CT showed an increase in DOTANOC avidity in the prostate gland, and his prostate biopsy specimen was stained positive for ACTH and markers for neuroendocrine differentiation. He was started on ketoconazole, which was switched to IV octreotide in view of liver dysfunction from hepatic metastases. He eventually succumbed to the disease after 3 months of his diagnosis. It is imperative to recognize prostate carcinoma as a source of ectopic ACTH secretion as it is associated with poor clinical outcomes, and the diagnosis can be missed due to atypical clinical presentations.

## 1. Introduction

Ectopic secretion of adrenocorticotropic hormone (ACTH) is responsible for approximately 10–20% of all causes of Cushing syndrome [1]. The classic sources of ectopic ACTH secretion include bronchial carcinoid tumours, small cell lung carcinoma, thymoma, medullary thyroid carcinoma (MTC), gastroenteropancreatic neuroendocrine tumours (NET), and phaeochromocytomas [2]. Ectopic adrenocorticotropic syndrome (EAS) is diagnostically challenging due to its variable clinical manifestations; however, prompt recognition and treatment is critical. Ectopic ACTH production from prostate carcinoma is rare, and there are less than 50 cases published to date. Here, we report a case of ectopic Cushing's syndrome secondary to prostate adenocarcinoma who did not present with the typical physical features of Cushing's syndrome, but instead with features of severe hypercortisolism such as hypokalaemia, oedema, and sepsis.

## 2. Case Presentation

A 61-year-old male presented to our institution with recurrent hypokalaemia, lower limb weakness, and oedema. He had a history of recently diagnosed metastatic prostate adenocarcinoma, for which he was started on leuprolide and finasteride. Other medical history includes poorly controlled diabetes mellitus and hypertension of 1-year duration. He presented with hypokalaemia of 2.7 mmol/L associated with bilateral lower limb oedema and weakness, initially attributed to the intake of complementary medicine, which resolved with potassium supplementation and cessation of the complementary medicine. One month later, he was readmitted for refractory hypokalaemia of 2.5 mmol/L and progression of the lower limb weakness and oedema. On examination, his blood pressure (BP) was 121/78 mmHg, and body mass index (BMI) was 24 kg/m^2^. He had no Cushingoid features of rounded and plethoric facies, supraclavicular or dorsocervical fat pad, ecchymoses, and no purple striae on the abdominal examination. He had mild bilateral lower limb proximal weakness and oedema.

His initial laboratory findings of severe hypokalaemia with metabolic alkalosis (potassium 2.5 mmol/L and bicarbonate 36 mmol/L), raised 24-hour urine potassium (86 mmol/L), suppressed plasma renin activity and aldosterone, central hypothyroidism, and elevated morning serum cortisol (1229 nmol/L) (Table 1) raised the suspicion for endogenous hypercortisolism. Furthermore, hormonal evaluations confirmed ACTH-dependent Cushing's syndrome with inappropriately normal ACTH (56 ng/L) and failure of cortisol suppression after 1 mg low-dose, 2-day low-dose, and 8 mg high-dose dexamethasone suppression tests (Table 2). His 24-hour urine free cortisol (UFC) was significantly elevated at 20475 (59–413) nmol/day.

**Figure 1:**
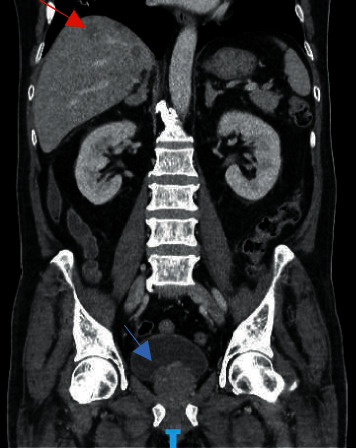
CT thorax abdomen and pelvis showing prostate cancer (blue arrow) with liver metastases (red arrow).

**Figure 2:**
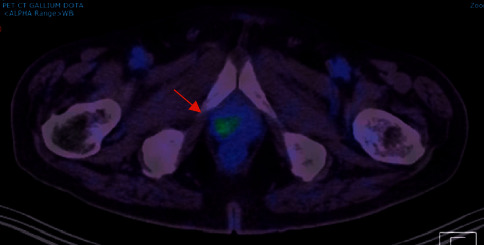
Ga68-DOTANOC PET/CT demonstrating increased DOTANOC avidity seen in the inferior aspect of the right side of the prostate gland (red arrow).

**Figure 3:**
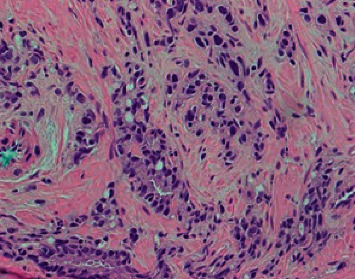
Hematoxylin and eosin staining showing acinar adenocarcinoma of the prostate featuring enlarged, pleomorphic cells infiltrating as solid nests and cords with poorly differentiated glands (Gleason score 5 + 4 = 9).

**Figure 4:**
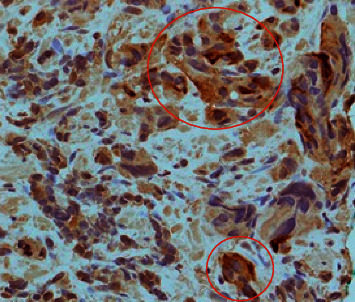
Positive ACTH immunohistochemical staining of prostate tumour (within the circle).

To identify the source of excessive cortisol secretion, magnetic resonance imaging (MRI) of the pituitary fossa and computed tomography (CT) of the thorax, abdomen, and pelvis were performed. Pituitary MRI was unremarkable, and CT scan showed the known prostate lesion with extensive liver, lymph nodes, and bone metastases (Figure 1). To confirm that the prostate cancer was the source of ectopic ACTH production, gallium-68 labelled somatostatin receptor positron emission tomography (PET)/CT (68Ga-DOTANOC) was done, which showed an increased DOTANOC avidity in the inferior aspect of the prostate gland (Figure 2). Immunohistochemical staining of his prostate biopsy specimen was requested, and it stained positive for ACTH and markers of neuroendocrine differentiation (synaptophysin and CD 56) (Figures 3 and 4), establishing the diagnosis of EAS secondary to prostate cancer.

**Figure 5:**
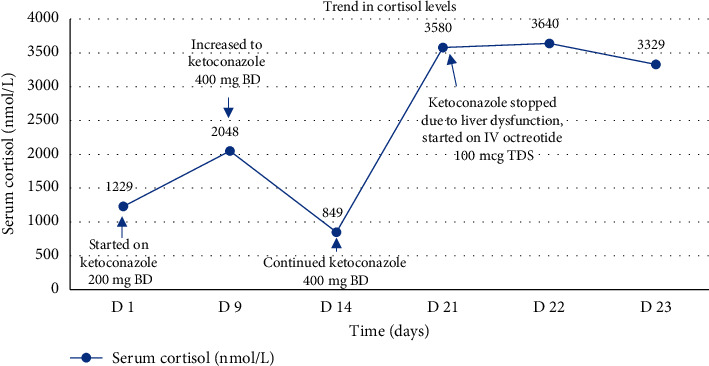
The trend in cortisol levels on pharmacological therapy.

The patient was started on potassium chloride 3.6 g 3 times daily and spironolactone 25 mg once daily with normalisation of serum potassium. His BP was controlled with the addition of lisinopril and terazosin to spironolactone and ketoconazole, and his blood glucose was well controlled with metformin and sitagliptin. To manage the hypercortisolism, he was treated with ketoconazole 400 mg twice daily with an initial improvement of serum cortisol from 2048 nmol/L to 849 nmol/L (Figure 5). Systemic platinum and etoposide-based chemotherapy was recommended for the treatment of his prostate cancer after a multidisciplinary discussion, but it was delayed due to severe bacterial and viral infection. With the development of liver dysfunction, ketoconazole was switched to intravenous octreotide 100 mcg three times daily as metyrapone was not readily available in our country. However, the efficacy was suboptimal with marginal reduction of serum cortisol from 3580 nmol/L to 3329 nmol/L (Figure 5). The patient continued to deteriorate and was deemed to be medically unfit for chemotherapy or bilateral adrenalectomy. He was referred to palliative care services, and he eventually demised due to cancer progression within 3 months of his diagnosis.

## 3. Discussion

Ectopic ACTH secretion is an uncommon cause of Cushing's syndrome accounting for approximately 9–18% of the patients with Cushing's syndrome [3]. Clinical presentation is highly variable depending on the aggressiveness of the underlying malignancy, but patients typically present with symptoms of severe hypercortisolism such as hypokalaemiaa, oedema, and proximal weakness which were the presenting complaints of our patient [4]. The classical symptoms of Cushing's syndrome are frequently absent due to the rapid clinic onset resulting in diagnostic delay [5].

Prompt diagnosis and localisation of the source of ectopic ACTH secretion are crucial due to the urgent need for treatment initiation. The usual sources include small cell lung carcinoma, bronchial carcinoid, medullary thyroid carcinoma, thymic carcinoid, and pheochromocytoma. CT of the thorax, abdomen, and pelvis should be the first-line imaging modality, and its sensitivity varies with the type of tumour ranging from 77% to 85% [6]. Functional imaging such as 18-fluorodeoxyglucose-PET and gallium-68 labelled somatostatin receptor PET/CT can be useful in localising the source of occult EAS, determining the neuroendocrine nature of the tumour or staging the underlying malignancy [3, 6]. As prostate cancer is an unusual cause of EAS, we proceeded with 68Ga-DOTANOC PET/CT in our patient to localise the source of ectopic ACTH production.

The goals of management in EAS include treating the hormonal excess and the underlying neoplasm as well as managing the complications secondary to hypercortisolism [3]. Prompt management of the cortisol excess is paramount as complications such as hyperglycaemia, hypertension, hypokalaemia, pulmonary embolism, sepsis, and psychosis can develop especially when UFC is more than 5 times the upper limit of normal [3]. Ideally, surgical resection is the first-line management, but this may not be feasible in metastatic, advanced, or occult diseases.

Pharmacological agents are frequently required with steroidogenesis inhibitors such as ketoconazole and metyrapone, which reduce cortisol production effectively and rapidly [3, 6], the main drawback of ketoconazole being its hepatic toxicity. The efficacy of ketoconazole is reported to be 44%, metyrapone 50–75%, and ketoconazole-metyrapone combination therapy 73% [3, 7]. Mitotane, typically used in adrenocortical carcinoma, is effective in controlling cortisol excess but has a slow onset of action [3, 8]. Etomidate infusion can be used for short-term rapid control of severe symptomatic hypercortisolism and can serve as a bridge to definitive therapy [9]. Mifepristone, a glucocorticoid receptor antagonist, is indicated mainly in difficult to control hyperglycaemia secondary to hypercortisolism [8]. Somatostatin analogue has been proposed as a possible pharmacological therapy due to the expression of somatostatin receptors by ACTH secreting tumours [8, 10]. Bilateral adrenalectomy should be considered in patients with severe symptomatic hypercortisolism and life-threatening complications who cannot be optimally managed with medical therapies, especially in patients with occult EAS or metastatic disease [3, 8]. Bilateral adrenalectomy results in immediate improvement in cortisol levels and symptoms secondary to hypercortisolism [11]. However, surgical complications, morbidity, and mortality are high in patients with uncontrolled hypercortisolism [8], and our patient was deemed by his oncologist and surgeon to have too high a risk for bilateral adrenalectomy. For the treatment of prostate carcinoma, platinum and etoposide-based chemotherapies have been used, but their efficacy is limited with a median survival of 7.5 months [4, 12]. The side effects of chemotherapy can be severe with an enhanced risk of infection due to both cortisol and chemotherapy-mediated immunosuppression. Prompt control of hypercortisolism prior to chemotherapy and surgical procedure is strongly suggested to attenuate life-threatening complications such as infection, thrombosis, and bleeding with chemotherapy or surgery as well as to improve prognosis [3, 13].

There are rare reports of ectopic ACTH secretion from prostate carcinoma. These tumours were predominantly of small cell or mixed cell type, and pure adenocarcinoma with neuroendocrine differentiation are less common [4, 5]. There is a strong correlation between the prognosis and the types of malignancy in patients with EAS, and patients with prostate carcinoma have a poor prognosis [4]. These patients had metastatic disease at presentation, and the median survival was weeks to months despite medical treatment, chemotherapy, and even bilateral adrenalectomy [4], as seen with our patient who passed away within 3 months of his diagnosis.

In conclusion, adenocarcinoma of the prostate is a rare cause of EAS. The diagnosis and management are complex and challenging requiring specialised expertise with multidisciplinary involvement. The presentation can be atypical, and it is imperative to suspect and recognise prostate carcinoma as a source of ectopic ACTH secretion. Prompt initiation of treatment is important, as it is a rapidly progressive and aggressive disease associated with intense hypercortisolism resulting in high rates of mortality and morbidity.

## Figures and Tables

**Table 1:** Investigations done during his 2^nd^ admission.

Renal panel	Value (normal range)
Urea	3.9 (2.7–6.9 mmol/L)
Sodium	143 (136–146 mmol/L)
Potassium	2.5 (3.6–5.0 mmol/L)
Chloride	94 (100–107 mmol/L)
Bicarbonate	36 (19.0–29.0 mmol/L)
Creatinine	41 (37–75 umol/L)
Estimated glomerular filtration rate	>90 (mL/min/1.73 m^2^)
24-hour urine potassium	86 mmol/L

Hormonal workup	
Plasma renin activity	<0.6 (0.6–3.0 ng/mL/h)
Aldosterone	<4.0 (≤21 ng/dL)
8 am cortisol	1229 nmol/L
Free thyroxine	5.5 (10–20 pmol/L)
Thyroid stimulating hormone	0.378 (0.4–4.0 mIU/L)

Others	
Prostate-specific antigen	89 (0.0–4.0 ug/L)
HbA1c	9.0%

**Table 2:** Diagnostic workup for hypercortisolism.

	Value (normal range)
8 am cortisol	1229 nmol/L
ACTH	56.0 (10–60 ng/L)
1 mg overnight dexamethasone suppression test	1327 nmol/L
2-day low-dose dexamethasone suppression test	1447 nmol/L
8 mg high-dose dexamethasone suppression test	1229 ≥ 1424 nmol/L
24-hour UFC	20475 (59–413 nmol/day)

## Data Availability

The data used to support the findings of this study are included within the article.
